# Impact of the visceral adipose tissue on bone quality in patients with untreated mild‐to‐severe obstructive sleep apnea

**DOI:** 10.1111/jsr.14397

**Published:** 2024-12-10

**Authors:** Giulia Sartori, Francesco Bertoldo, Andrea Gretter, Federica Margherita Lovati, Rosaria Caprino, Giovanni Viterale, Ernesto Crisafulli

**Affiliations:** ^1^ Department of Medicine, Respiratory Medicine Unit University of Verona and Azienda Ospedaliera Universitaria Integrata of Verona Verona Italy; ^2^ Emergency Medicine Unit, Department of Medicine University of Verona and Azienda Ospedaliera Universitaria Integrata of Verona Verona Italy

**Keywords:** fat distribution, obstructive sleep apnea, trabecular bone score, vertebral fractures, visceral adipose tissue

## Abstract

Obstructive sleep apnea (OSA) predominantly affects patients who are obese and causes systemic organ damage. Little is known about the relationship between fat distribution and bone impairment in these patients. We aimed to evaluate the impact of the visceral adipose tissue (VAT) on the bone quality of patients with OSA. In our prospective study, 49 untreated patients with mild‐to‐severe OSA underwent dual‐energy X‐ray absorptiometry. Polygraphy data were also collected. According to the recent reference values for European adults, patients were divided by the sex‐related threshold of the VAT index into two categories: VAT index within limits (normal VAT [nVAT]) and increased VAT (iVAT). In all, 63% of the patients were in the iVAT category. Compared to patients with nVAT, those with iVAT had a higher prevalence of arterial hypertension (52% versus 22%) and diabetes (32% versus 6%), and higher values of mean nocturnal desaturation. Patients with iVAT had, in comparison to those with nVAT, lower values of the lumbar spine trabecular bone score (TBS; mean 1.24 versus 1.39; *p* < 0.001), TBS T‐score (mean −1.82 versus −0.52; *p* < 0.001) and TBS Z‐score (mean −0.35 versus 0.75; *p* = 0.002). Moreover, a close association was present between the VAT index and TBS lumbar spine L1–L4 (*r* ^2^ linear 0.573; *p* < 0.001), and altered values of the TBS Z‐score were associated with the severity of vertebral fractures. Finally, in a linear regression‐adjusted model, the VAT index predicted TBS lumbar spine L1–L4 (*β* −0.323; *p* < 0.001). In patients with OSA VAT impacts bone quality. In these patients, the role of VAT as a metabolically active tissue should be considered.

## 1 INTRODUCTION

Obstructive sleep apnea (OSA) is characterised by recurrent sleep‐related airflow cessation during sleep, causing apneic events (Lévy et al., 2015); these respiratory events may result in intermittent hypoxaemia, autonomic fluctuation, and sleep fragmentation. Patients with OSA have an elevated risk of hypertension and cardiovascular (CV) disease (Salman et al., 2020); several pathophysiological factors contribute to the relationship between OSA and vascular risk, including neurohormonal dysregulation, endothelial dysfunction, and inflammation (Salman et al., 2020). In patients with OSA, the related hypoxic burden sign of nocturnal intermittent hypoxaemia has been recognised as a potential risk factor for CV events and all‐cause mortality (Trzepizur et al., 2022). Therefore, variables of oxygen desaturation rather than apnea indexes were robust predictors of CV events (Xu et al., 2023).

Obesity leads to a chronic inflammatory state, significantly increasing the risk of CV disease (Rana & Neeland, 2022). Obesity constitutes the primary risk factor for developing OSA (Carter & Watenpaugh, 2008), mainly visceral (Gaines et al., 2018), strongly associated with the severity of apnea, especially in males (Kritikou et al., 2013). Measures of visceral adipose tissue (VAT) may identify subjects with elevated cardiometabolic risk factors (Katzmarzyk et al., 2013). For this reason, there is great interest in the mechanisms linking OSA and the metabolic function of VAT (Ryan, 2017). Intermittent hypoxaemia induces a pro‐inflammatory phenotype of the VAT with the polarisation of adipose tissue macrophages towards an M1‐pro‐inflammatory subtype, upregulation and secretion of numerous pro‐inflammatory adipokines and subsequent impairment of the insulin‐signalling pathway (Ryan, 2017). Although VAT is currently measured by using computed tomography or magnetic resonance imaging (Fang et al., 2018), dual‐energy X‐ray absorptiometry (DXA) can accurately measure body composition with high precision and low X‐ray exposure, assessing more readily in clinical settings (Kaul et al., 2012).

Although plausible, the evidence supporting the association between OSA and bone alterations is inconsistent, with some risks of bias (Eimar et al., 2017). Compared to non‐OSA subjects, males with severe OSA have a significantly lower bone mineral density (BMD) in the lumbar spine, while OSA and BMD had no association in females (Hamada et al., 2016). In elderly subjects, on the contrary, OSA is associated with higher BMD, with oxygen desaturations being a significant determinant of bone metabolism, suggesting an active role of intermittent hypoxaemia in stimulating of the bone remodelling process (Sforza et al., 2013). A recent prospective study considering young patients with OSA has documented the relationship between bone metabolism and nocturnal hypoxaemia status (Zhao et al., 2022). Low oxygen saturation was an independent explanatory variable for lumbar BMD (Zhao et al., 2022). Relatively, a recent report on severe male OSA observed a significant increase in BMD lumbar spine after continuous positive airway pressure (CPAP) treatment (Carpi et al., 2024).

The trabecular bone score (TBS) is a grey‐level textured measure derived from spine DXA images, representing an indirect and non‐invasive measure of trabecular bone microarchitecture with a potential clinical utility in predicting fracture risk (Silva & Leslie, 2017). A low TBS indicates degraded bone microarchitecture, predicts osteoporotic fracture, and is an independent risk factor for fracture, actually included in the Fracture Risk Assessment Tool (FRAX), the most clinically used fracture risk calculation algorithm (Leslie et al., 2023). In patients with diabetes mellitus, there is a complex relationship between OSA severity and TBS, with more severe OSA predicting lower TBS in men but predicting higher TBS in postmenopausal women (Nimitphong et al., 2019). Moreover, in patients with diabetes mellitus, a reduction in visceral fat may be related to the improvement in the TBS (Moon et al., 2020), also a hypothesis in patients with OSA in which an active interconnection between VAT and bone quality is not yet known. Moreover, although in a population‐based study, visceral adiposity was associated with lung function impairment (He et al., 2021), we assume an impact of the VAT on nocturnal desaturations of patients with OSA and consequently on bone. Also, this last aspect has yet to be encountered in the literature. Therefore, our study aimed to examine whether the VAT is related to bone quality variables.

## 2 METHODS

### 2.1 Study cohort

From September 2021 to October 2022, we prospectively considered any subjects admitted for a suspected nocturnal respiratory disorder to a dedicated ambulatory clinic of the Respiratory Medicine Unit of the Azienda Ospedaliera Universitaria Integrata of Verona. Then, we selected and considered patients with OSA of any grade for the study. Subjects with normal polygraphy or with another disease (e.g., obesity hypoventilation syndrome [OHS]), patients with OSA who received any treatment for OSA (e.g., CPAP), patients unable to undergo a DXA (e.g., a body weight >120 kg, the limit for the support of the DXA scanner), or unavailable to undergo a DXA, or patients in whom all data related to respiratory polygraphy or DXA were missing were excluded. The Institutional Review Board of the Department of Medicine of the University of Verona (Italy) approved the study, which was conducted according to the Good Clinical Practice and the Helsinki Declaration statements.

**FIGURE 1:**
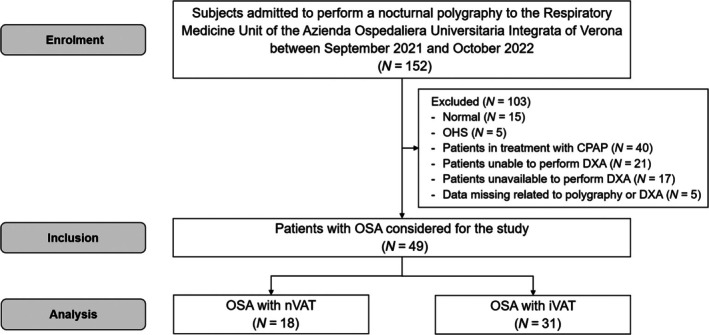
Study flow chart. CPAP, continuous positive airway pressure; DXA, dual‐energy X‐ray absorptiometry; OHS, obesity hypoventilation syndrome; OSA, obstructive sleep apnea; VAT, visceral adipose tissue.

Figure 1 shows the study flow diagram.

### 2.2 General measures

We collected demographic and anthropometric characteristics (body mass index [BMI]), including body circumferences (neck, waist, and hip). In addition, data related to the main comorbidities of arterial hypertension, heart disease, diabetes, peripheral arterial disease, and asthma/chronic obstructive pulmonary disease were obtained from self‐administered questionnaires.

Lifestyle information included smoking habits and the related number of packs/years.

Finally, patients completed the Italian version of the Epworth Sleepiness Scale (ESS), a self‐report questionnaire to assess daytime sleepiness (Vignatelli et al., 2003). The ESS consists of eight items measuring the probability of falling asleep during daily life situations, and the total score can range from 0 to 24 (Vignatelli et al., 2003).

### 2.3 Nocturnal polygraphy

Respiratory polygraphy with a portable Nox T3s™ device (https://noxmedical.com, USA) was used. The following signals were detected: blood oxygen saturation (by a finger sensor), thoracic and abdominal movements (by inductive belts), nasal airflow (derived from the belts), snoring and body position (both detected by the device). This portable device has demonstrated very good measurement agreement compared to in‐laboratory polysomnography and a high sensitivity for detecting even mild OSA (Cairns et al., 2014). The device's signals were analysed using the Noxturnal™ sleep study software, which uses an advanced automated scoring algorithm for respiratory analysis.

The polygraphy reports, analysed according to the American Academy of Sleep Medicine document (Sateia, 2014), included the total sleep time in minutes (TST), the apnea–hypopnea index (AHI) calculated as the number of apneas and hypopneas in an hour (events/h), parameters related to pulse oximetry oxygen saturation (SpO_2_), in particular the mean and lowest SpO_2_, the number of events in an hour of oxygen desaturation of 3% (Sforza et al., 2013), the mean of desaturation, and the sleep time evaluated in minutes with SpO_2_ <90% (ST90). An apnea was considered as absent airflow for ≥10 s, while a hypopnea was a decrease in airflow (>30%) for ≥10 s associated with oxygen desaturation (≥3%). The AHI was also considered as a categorical variable, defining the mild (AHI ≥5 and ≤15 events/h), moderate (AHI >15 and ≤30 events/h), and severe (AHI >30 events/h) stages (Lévy et al., 2015).

To exclude the presence of OHS among patients with OSA, we considered patients who were obese (BMI ≥40 kg/m^2^), the predictor of ST90 >30%, as reported by Pıhtılı et al. (2017). Then, in patients having these characteristics, we performed an arterial blood gas analysis to confirm the chronic hypoventilation reported during wakefulness as an increase of partial pressure of arterial carbon dioxide ≥45 mmHg or serum bicarbonate ≥27 mmol/L (Masa et al., 2019).

We considered only patients with a recorded polygraphy report with good signal quality.

### 2.4 Fat and bone assessment

All total body and lumbar spine DXA scans were performed using the same device (Discovery QDR A System—Hologic, Inc., Waltham, MA, USA). Data on visceral fat mass, android fat mass, android lean mass (Gregori et al., 2023), android soft tissue mass, and android fat percentage were derived from the total body DXA scan. All scan images were reviewed, and any with excessive movement or artefact were excluded. The DXA instrument underwent daily calibration using a spine phantom. Whole‐body measurements were calibrated using a phantom.

The VAT was measured as an absolute value, and according to recent reference values of VAT DXA‐derived index (VAT index), it is calculated from the European adult population (Lundblad et al., 2021). Therefore, according to the sex‐related threshold values of the VAT index (Lundblad et al., 2021), we divided patients into two categories: VAT index within limits (normal VAT [nVAT]) and increased VAT (iVAT).

Site‐matched TBS parameters were extracted from the lumbar spine DXA image using softwareTBSiNsight® (Medimaps Group, Geneva, Switzerland). The most recent version of the TBS software (version 4.0) is based on a computational algorithm that considers the individual's regional soft tissue thickness instead of its surrogate BMI and considers bone architecture variables. In the DXA, the algorithm acquires and re‐processes the variations of the grey scale related to the attenuation of the X‐ray in the different pixels of a trabecular structure, particularly in the image of the lumbar spine (L1–L4). Moreover, we used three TBS cut‐off values, in particular, ≥1.350, 1.200–1.350 and <1.200, as signs of normal, partially degraded, and degraded bone microarchitecture, respectively. TBSs for age and gender (TBS Z‐score) acquired from the manufacturer's (TBS iNsight) reference database (only available for females ≥45 years and males ≥40 years of age) were estimated (Leslie et al., 2023).

When available, we took lateral chest X‐rays from patients in the last year to perform a morphometric assessment of prevalent vertebral fractures from D2 to L1. The morphometric evaluation of the vertebral fractures was based on the semiquantitative method proposed by Genant et al. (1993). This method provides an overview of the severity of a fracture, which is assessed solely by visual estimation of the extent of vertebral height reduction and morphological change of the vertebra. In Genant et al. (1996) semiquantitative visual assessment, each vertebra receives a severity grade based on the visually apparent degree of vertebral height loss. According to the visual inspection, the levels of vertebral alteration are Grade 0, mildly deformed (Grade 1: 20–25% reduction in height and 10–20% reduction in projected vertebral area); moderately deformed (Grade 2: 26–40% reduction in height and 21–40% reduction in projected vertebral area); severely deformed (Grade 3: >40% reduction in height and projected vertebral area). Finally, we assess the spinal deformity index (SDI; Kerkeni et al., 2009), which was calculated by summing the grade of each vertebra from T2 to L1 for each patient. The SDI is an expression of the severity of fragility and is a good predictor of incident vertebral fractures (Kerkeni et al., 2009).

### 2.5 Statistical analysis

Due to the study's exploratory design, a sample size calculation was executed. A preliminary Shapiro–Wilk test was performed. Data are reported as percentages for categorical variables and mean (± standard deviation [SD]) or median (interquartile range) for continuous variables. Categorical variables were compared by the chi‐squared test or the Fisher's exact test, while the independent *t*‐test or the non‐parametric Mann–Whitney *U* test assessed continuous variables.

Relationships between variables were assessed using the Pearson correlation coefficient (*r* and *r* ^*2*^). Multivariate regression linear models (method: enter) were performed to identify variables predicting the TBS lumbar spine L1–L4, considered dependent variables for each model. Variables that showed a significant result were included in the model; to avoid collinearity, strongly correlated variables (*r* > | ± 0.4|) were excluded from the multivariate analyses. The beta (*β*), standard error (SE), and 95% confidence interval (CI) for *β* were calculated for each model.

All analyses were performed using IBM Statistical Package for the Social Sciences (SPSS), version 17.0 (IBM Corp., Armonk, NY, USA), and a *p* < 0.05 was considered to indicate statistical significance.

## 3 RESULTS

Of the 49 patients considered, 31 had iVAT. In comparison to patients with nVAT (Table 1), those with iVAT had higher BMI values and all measured circumferences (neck, waist, and hip) in addition to a higher prevalence of arterial hypertension (52% versus 22%) and diabetes (32% versus 6%). Moreover, patients with iVAT, compared to those with nVAT, had higher values for mean nocturnal desaturation (median 4.6% versus 3.7%; *p* = 0.002) and lower values of TBS lumbar spine L1–L4 (mean 1.24 versus 1.39), TBS lumbar spine T‐score (mean −1.82 versus −0.52), TBS lumbar spine Z‐score (mean −0.35 versus 0.75).

**TABLE 1:** General characteristics according to the level of the visceral adipose tissue.

Variable	OSA with nVAT^a^ (*n* = 18)	OSA with iVAT^a^ (*n* = 31)	*p*
Age, years, median (IQR)	57 (11.5)	64 (22)	0.085
Male, *n* (%)	16 (89)	27 (87)	>0.999
BMI, kg/m^2^, median (IQR)	26.4 (5.2)	35.8 (7.8)	**<0.001**
Obesity (BMI ≥30 kg/m^2^), *n* (%)	4 (22)	25 (81)	**<0.001**
Circumferences, cm			
Neck, mean (SD)	39.8 (3.6)	44.0 (4.1)	**0.002**
Waist, mean (SD)	100.7 (14.6)	119.6 (14.6)	**<0.001**
Hip, median (IQR)	104 (11.5)	119 (22)	**<0.001**
Smoking habit, *n* (%)			
No	7 (39)	18 (58)	0.211
Former	6 (33)	10 (32)	
Current	5 (28)	3 (10)	
Pack/year, median (IQR)	12 (11.6)	22.5 (27)	0.090
Arterial hypertension, *n* (%)	4 (22)	16 (52)	**0.044**
Heart disease, *n* (%)	6 (33)	11 (35)	0.879
Diabetes, *n* (%)	1 (6)	10 (32)	**0.038**
Peripheral arterial disease, *n* (%)	2 (11)	3 (10)	>0.999
Asthma/COPD, *n* (%)	2 (11)	3 (10)	>0.999
VAT, g, mean (SD)	681.7 (187.4)	1392.2 (295.2)	**<0.001**
VAT index, mean (SD)	0.39 (0.10)	0.81 (0.17)	**<0.001**
Epworth Sleepiness Scale score, median (IQR)	7 (7.5]	5 (4]	0.093
TST, min, median (IQR)	464 (14)	472 (41)	0.573
AHI, events/h, median (IQR)	19.2 (22.2)	18.3 (15.2)	0.449
AHI stages, *n* (%)			
Mild	7 (39)	14 (45)	0.302
Moderate	4 (22)	11 (36)	
Severe	7 (39)	6 (19)	
Mean SpO_2_, %, median (IQR)	93.2 (2.9)	92.5 (3.7)	0.678
Lowest SpO_2_, %, median (IQR)	81 (8.5)	82 (11)	0.771
ODI (3%), events/h, median (IQR)	18 (24.7)	19.8 (16.6)	0.740
Mean of desaturations, %, median (IQR)	3.7 (0.7)	4.6 (2.6)	**0.002**
ST90, min, median (IQR)	7.2 (40.6)	29 (85.5)	0.070
ST90, %, median (IQR)	1.5 (10.8)	6.05 (19.02)	0.067
TBS lumbar spine L1–L4, mean (SD)	1.39 (0.11)	1.24 (0.12)	**<0.001**
TBS lumbar spine T‐score, mean (SD)	−0.52 (0.93)	−1.82 (1.00)	**<0.001**
TBS lumbar spine Z‐score, mean (SD)	0.75 (0.95)	−0.35 (1.21)	**0.002**
BMD femur, mean (SD)	0.96 (0.14)	1.03 (0.14)	0.079
BMD femur T‐score, mean (SD)	−0.47 (0.87)	0.08 (0.88)	**0.041**
BMD femur Z‐score, mean (SD)	0.094 (0.70)	0.75 (0.87)	**0.010**

*Note*: The data are shown as the number of patients (%) or median (interquartile range [IQR], 25th–75th percentiles). Percentages are calculated for non‐missing data. Bold values statistically significant at *p* < 0.05.

Abbreviations: AHI, apnea–hypopnea index; BMD, bone mineral density; BMI, body mass index; COPD, chronic obstructive pulmonary disease; ODI, oxygen desaturation index; OSA, obstructive sleep apnea; SpO_2_, pulse oximetry oxygen saturation; ST90, sleep time with oxygen saturation <90%; TBS, trabecular bone score; TST, total sleep time; (i)(n)VAT, (increased) (normal) visceral adipose tissue.

^a^ Categories were divided according to the VAT index's sex‐related threshold values, derived from the Tromsø Study 2015–2016 (Lundblad et al., 2021).

**FIGURE 2:**
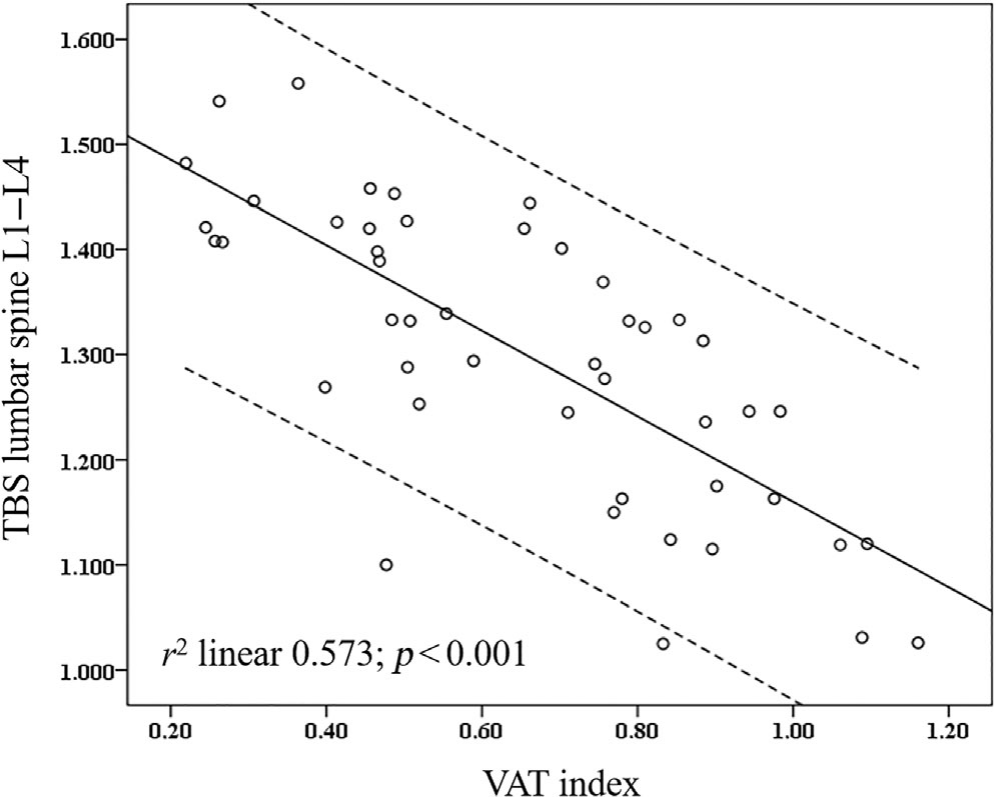
Scatterplot between VAT and TBS lumbar spine L1–L4. TBS, trabecular bone score; (i)(n)VAT, (increased) (normal) visceral adipose tissue.

Table 2 shows the correlation analyses between VAT, sleep, and bone‐related variables. The VAT index was strongly correlated with mean desaturation (*r* 0.424; *p* < 0.01), TBS lumbar spine L1–L4 (*r* −0.757; *p* < 0.001), TBS lumbar spine T‐score (*r* −0.749; *p* < 0.001), TBS lumbar spine Z‐score (*r* − 0.634; *p* < 0.001). The *r* ^2^ between the VAT index and TBS lumbar spine L1–L4 was strongly significant (0.573; *p* < 0.001) (Figure 2).

**TABLE 2:** Correlation analyses concerning the visceral adipose tissue.

		1	2	3	4	5	6	7	8	9	10	11
1	VAT index	‐										
2	AHI	0.120	‐									
3	Lowest SpO_2_	−0.258	−0.498***	‐								
4	ODI (3%)	0.164	0.899***	−0.555***	‐							
5	Mean of desaturation	0.424**	0.082	−0.247	0.066	‐						
6	ST90, min	0.169	0.154	−0.442**	0.196	0.594***	‐					
7	TBS LS L1–L4	−0.757***	−0.056	0.146	−0.128	−0.148	−0.037	‐				
8	TBS LS T‐score	−0.749***	−0.026	0.111	−0.103	−0.147	−0.060	0.991***	‐			
9	TBS LS Z‐score	−0.634***	−0.041	0.157	−0.162	−0.049	−0.011	0.939***	0.928***	‐		
10	BMD F	0.270	0.010	−0.201	0.049	0.201	0.019	−0.072	−0.039	−0.158	‐	
11	BMD F T‐score	0.262	−0.034	−0.163	0.011	0.189	0.024	−0.046	−0.035	−0.133	0.980***	‐
12	BMD F Z‐score	0.303*	−0.061	−0.091	−0.048	0.265	0.032	0.009	−0.005	0.040	0.859***	0.911***

*Note*: values are reported as Pearson *r*. Bold text values showed statistically significant correlations at the *p < 0.05, **p < 0.01 and ***p < 0.001 levels.

Abbreviations: AHI, apnea–hypopnea index; BMD, bone mineral density; F, femur; LS, lumbar spine; ODI, oxygen desaturation index; ST90, sleep time with oxygen saturation <90%; TBS, trabecular bone score; VAT, visceral adipose tissue.

Table 3 shows a linear regression model with the TBS lumbar spine L1–L4 as dependent variables, adjusted for age, sex, severity of OSA, and presence of diabetes and obesity. In this model, the VAT index predicts the TBS lumbar spine L1–L4 (*β* −0.323; 95% CI −0.48 to −0.16; *t* −4.15; *p* < 0.001).

**TABLE 3:** Linear regression models predicting bone quality.

Dependent variable: TBS lumbar spine L1–L4	Unadjusted model					Adjusted model^a^				
Independent variable	*β*	SE	95% CI for *β*	*t*	*p*	*β*	SE	95% CI for *β*	*t*	*p*
VAT index	−0.396	00.056	−0.509 to −0.283	−7.04	**<0.001**	−0.323	0.078	−0.48 to −0.16	−4.15	**<0.001**
ESS	0.002	00.004	−0.005 to 0.009	0.55	0.588	0.003	0.003	−0.004 to 0.010	0.83	0.411
Model summary	*r* = 0.759; *r* ^2^ = 0.576; adjusted *r* ^2^ = 0.557; SE = 0.091; *p* < 0.001					*r* = 0.823; *r* ^2^ = 0.677; adjusted *r* ^2^ = 0.628; SE = 0.083; *p* < 0.001				

*Note*: Bold text indicates a statistically significant difference.

Abbreviations: CI, confidence interval; ESS, Epworth Sleepiness Scale; TBS, trabecular bone score; SE, standard error; VAT, visceral adipose tissue.

^a^ Models were adjusted according to the age, sex, severity of obstructive sleep apnea (apnea–hypopnea index) and presence of obesity (body mass index ≥30 kg/m^2^) and diabetes.

**FIGURE 3:**
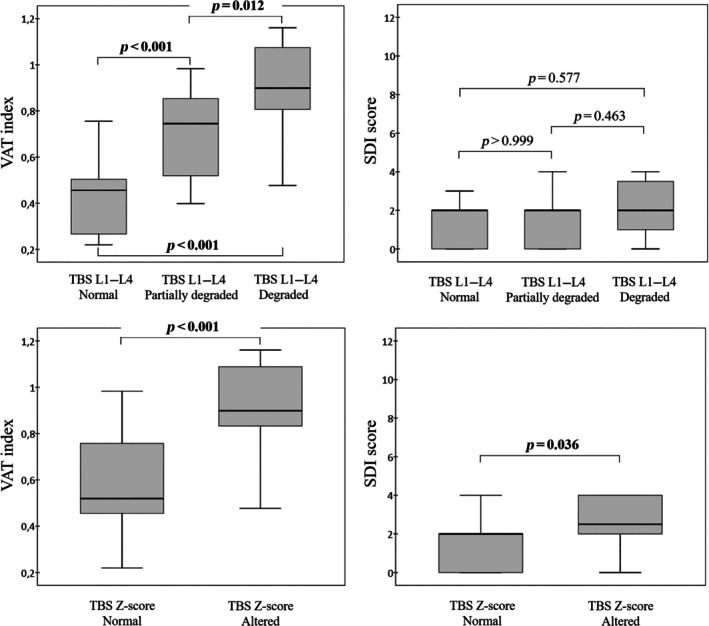
Boxplots concerning the bone microarchitecture alterations and the vertebral fractures. SDI, spine deformity index; TBS, trabecular bone score; VAT, visceral adipose tissue.

Figure 3 shows the boxplot distribution of the VAT index and SDI score according to the two methods of representing the bone microarchitecture alterations, according to three (TBS L1–L4 normal, partially degraded, and degraded) or two cut‐offs (TBS Z‐score normal or altered). While the VAT index was progressively and significantly distributed in any categories reported, the SDI was significantly distributed between the TBS Z‐score normal (mean [SD] 1.62 [1.76]) and altered (mean [SD] 4.33 [5.39]) categories (*p* = 0.036).

**FIGURE 4:**
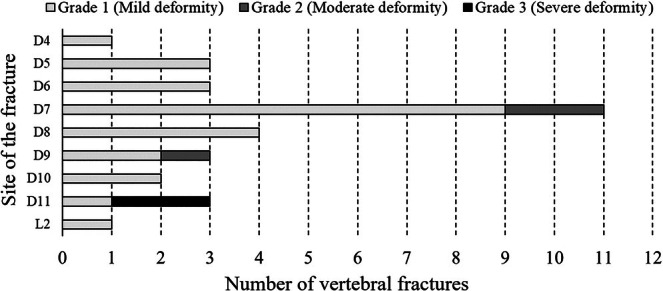
Prevalence of vertebral fractures and related sites and grades.

**FIGURE 5:**
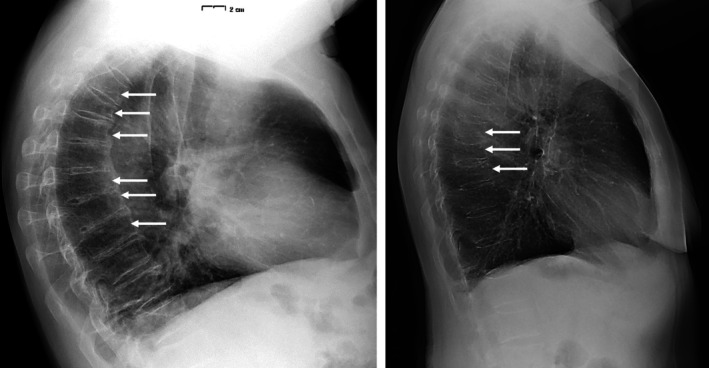
Examples of lumbar spine fractures evaluated by lateral view chest X‐ray.

**FIGURE 6:**
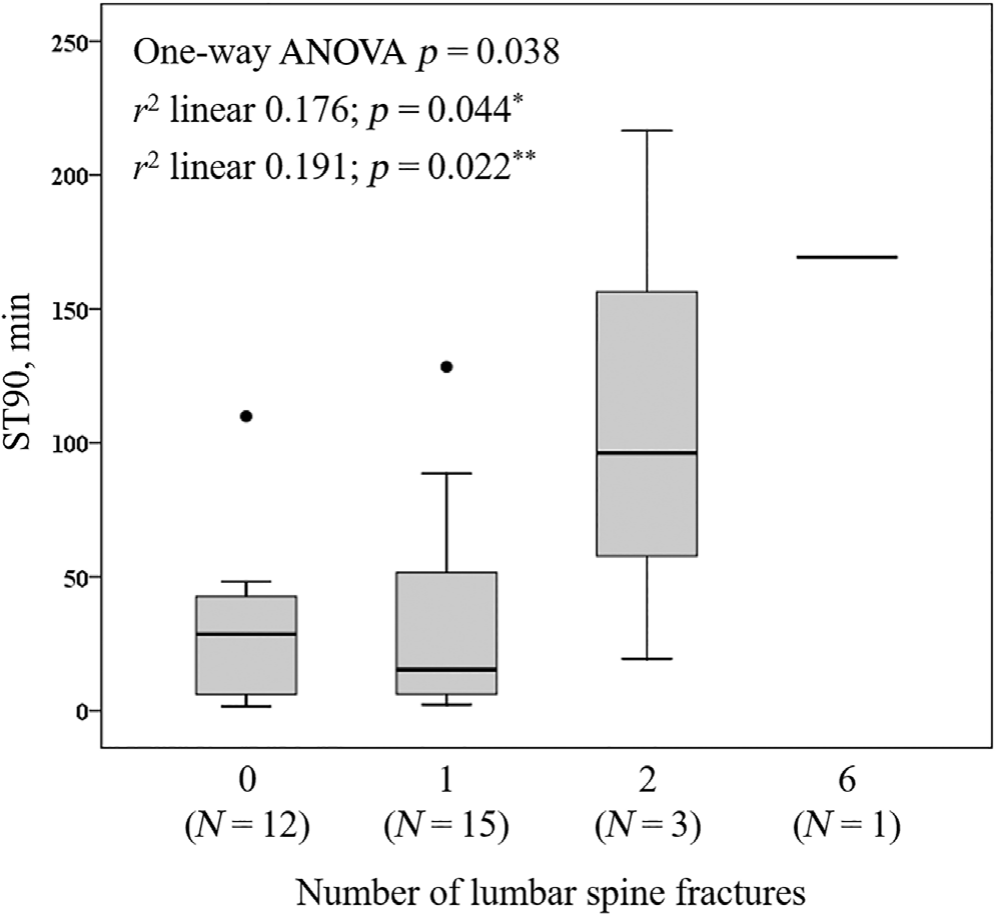
Distribution of ST90 (min) and number of lumbar spine fractures. Data were analysed in patients with lateral chest X‐rays and males only (*N* = 31). *Adjusted according to the presence of obesity (body mass index ≥30 kg/m^2^); **Adjusted according to the presence of visceral adipose tissue (VAT) categories (normal VAT and increased VAT). ANOVA, analysis of variance; ST90, sleep time with oxygen saturation <90%.

Figure 4 shows the distribution in the thoracic spine of the prevalent vertebral fractures and related grades. In all, 33 patients had lateral chest X‐rays. Respectively, one patient had six vertebral fractures, one patient had three fractures, three patients had two fractures, and 16 patients had one fracture for a total of 31 fractures. The SDI range was from 0 to 15 (mean [SD] 2.06 [2.87]), with 26, three, and two patients having fractures of Grade 1, 2, and 3, respectively. Examples of lumbar spine fractures evaluated by the lateral chest X‐ray are shown in Figure 5. No patient was symptomatic for fracture, and none was aware of having one or even more vertebrae fractures. Considering male patients only with lateral chest X‐rays (*N* = 31), the distribution of ST90 (min) was significantly different according to the number of lumbar spine fractures (Figure 6); moreover, there was a significant association between the two parameters (ST90 and fractures), also if adjusted according to the presence of obesity (BMI ≥30 kg/m^2^; *r* ^2^ linear 0.176, *p* = 0.044) and the VAT categories (nVAT and iVAT; *r* ^2^ linear 0.191, *p* = 0.022). There was no other association between respiratory and bone variables.

## 4 DISCUSSION

In a cohort of untreated patients with OSA, we demonstrated an active role of VAT in the alteration of the bone microarchitecture of the lumbar spine, involving a TBS L1–L4 reduction. Altered values of the TBS Z‐score were associated with vertebral fractures.

### 4.1 Impact of the visceral fat on patients with OSA

Once thought dormant and innocuous, the adipocyte emerges as a dynamic and influential cell (Shin et al., 2020). The adipose tissue microenvironment has a complex and heterogeneous cellular composition that also includes the adipose tissue‐derived stem and stromal cells (ASCs) and endothelial cells (Shin et al., 2020), communicating by numerous mediators, including adipokines, important link molecules between the nutritional state of the organism and the regulation of energy balance (Shin et al., 2020). Obesity triggers alterations in the ASCs, compromising the remodelling of adipose tissue and inducing low‐grade systemic inflammation, progressive insulin resistance, and other metabolic disorders (Favaretto et al., 2022). The pro‐inflammatory activity of the visceral tissue in patients with OSA is also inducted by intermittent nocturnal hypoxaemia (Ryan, 2017), representing a potential additional risk factor for CV events and all‐cause mortality in patients with OSA (Trzepizur et al., 2022). Of note, the VAT is closely associated with elevated cardiometabolic risk factors among adult subjects, independently of OSA presence (Katzmarzyk et al., 2013).

There is a strong association between visceral adiposity and OSA in terms of risk factors (Gaines et al., 2018) and disease severity (Kritikou et al., 2013). Although the presence of obesity, evaluated by BMI, is an independent risk factor for severe OSA to exhibit nocturnal oxygen desaturation (Deflandre et al., 2017) and intermittent nocturnal hypoxia is associated with metabolic risk in obese youth, of which only 23% were OSA (Narang et al., 2018), in this context, our study reports complementary findings concerning the significant relationship between the fat distribution in terms of VAT index and the mean of desaturations (*r* 0.424; *p* = 0.002; Table 2).

### 4.2 Impact of the visceral fat on bone quality

Obesity is a risk factor for fractures at different skeletal sites (Gonnelli et al., 2014) despite normal or high BMD (Rinonapoli et al., 2021), highlighting the skeletal fragility (bone quality) rather than quantity. Potential factors predisposing to fractures in adults with obesity include sarcopenia associated with obesity and impaired bone quality (Gregori et al., 2023). Lower muscle mass and strength and higher fat mass may impair bone quality (Gregori et al., 2023).

Studies have shown that the TBS negatively correlates with BMI or body weight, whereas BMD and bone biomechanical properties positively correlate with BMI or body weight (Langsetmo et al., 2016). This negative correlation between the TBS and BMI or weight could be due to the negative effect of excess fat on bone microarchitecture or a technical limitation of TBS due to the regional soft tissue noise in the DXA image. The TBS algorithm is probably limited because other microarchitectural parameters positively correlate with BMI. Despite this limitation, the TBS had excellent clinical performance (McCloskey et al., 2016).

Many investigators have shown that bone marrow adipose tissue expresses adipokines that can suppress bone formation or drive bone resorption by releasing cytokines that enhance receptor activator of nuclear factor‐κΒ ligand (RANKL) expression, which drives osteoclastogenesis. Also, it has been shown that marrow adipocyte‐like progenitors (Yu et al., 2021) directly release RANKL and interleukin 6, an inflammatory protein that may suppress osteoblast activity (Yu et al., 2021; Veldhuis‐Vlug & Rosen, 2018).

In obesity, adipose tissue grows and recruits new ASCs, developing ectopically and involving organs such as muscle, bone marrow, and heart (Favaretto et al., 2022). A possible explanation of the ectopic adipose organ origin may be related to stimulating adipogenic differentiation of mesenchymal precursors (Pillon et al., 2021). Interestingly, in obesity, the marrow adipose tissue (Veldhuis‐Vlug & Rosen, 2018) is positively associated with ectopic fat deposition, serum lipid levels, and VAT; it also increases with ageing in people with osteoporosis (Favaretto et al., 2022). Visceral and ectopic fat in the liver, muscles, or heart can increase the risk of developing insulin resistance, type 2 diabetes mellitus, and CV disease (Longo et al., 2019). In sarcopenic obesity, the increased presence of fat within the skeletal muscle causes a structural and functional impairment of the muscle, which can influence bone density, leading to osteoporosis. For example, myostatin produced by muscle tissue is reduced following fat infiltration into the muscles, which negatively regulates bone mass (McPherron et al., 1997). Osteocalcin (OCN; Mera et al., 2016) is a protein secreted in carboxylated form (cOCN) by osteoblasts and is responsible for matrix mineralisation and bone formation, while the subcarboxylated form (ucOCN) controls numerous physiological processes (Ducy et al., 1996). Overweight people and people with obesity have a reduced ucOC/OCN ratio, and this is known to be negatively related to bone quality.

On these bases, it is unsurprising that VAT is an independent predictor of bone turnover in women with obesity (Sharma et al., 2020), which determines profound alterations in the trabecular microarchitecture. In women with vertebral osteoporotic fracture, multivariate analysis revealed that higher visceral fat remained an independent predictor of vertebral fracture in patients who were overweight and obese (Zhang et al., 2022).

### 4.3 Impact of the visceral fat on bone quality of patients with OSA

In patients with OSA, the relationship between obesity and bone density is controversial (Eimar et al., 2017; Hamada et al., 2016; Sforza et al., 2013). TBS measures represent an indirect and non‐invasive measure of bone health (Silva & Leslie, 2017), with low TBS values being a sign of a degraded bone microarchitecture causing a risk of fractures, partially independent of bone density (BMD) (Leslie et al., 2023). Data about the TBS in patients with OSA are confused by the analysis performed in a cohort of patients with diabetes mellitus with values of lower or higher TBS (Nimitphong et al., 2019). For the first time, our study demonstrates the close and robust association between VAT and the TBS (Tables 2 and 3, Figure 2) when patients with OSA with iVAT have significantly lower TBS lumbar spine values (Table 1). Of note, the BMD femur T‐score and Z‐score were higher in iVAT, confirming the different aspects between microarchitecture (quality) and density (BMD) of the bone district analysed (Table 1). No nocturnal parameters were correlated with bone parameters.

Finally, we highlight the clinical role of our data related to vertebral fractures. Although no patient was aware of a vertebrae fracture, we identified and measured the parameters of 31 fractures in 33 patients (Figures 4 and 5); in one patient, we identified six vertebral fractures. Moreover, related to the mechanism reported above, we demonstrated the progressive association between VAT index and TBS degraded/altered, which is the last cause of vertebral fractures regarding SDI (Figure 3).

We found in a reduced number of male patients who had lateral chest X‐rays (*N* = 31), a significant association between the ST90 and the number of vertebral fractures (Figure 6). No other association was found between the same ST90 with bone quality variables or other respiratory and bone variables. Although we do not know the real cause of this association and any consideration needs to be done with caution due to the minimal sample, we may speculatively hypothesise it to be another hypoxic burden sign of nocturnal intermittent hypoxaemia (Trzepizur et al., 2022), of which the ST90 rather than AHI may represent a robust predictor (Xu et al., 2023). This persisting association appears exciting if adjusted for obesity or VAT categories and may open the door to future studies exploring the metabolic or hypoxaemic role in patients with OSA without an increased visceral fat or, in general, without obesity.

### 4.4 Strengths and limitations

Our strengths points are related to the possibility of exploring, for the first time in untreated patients with OSA, aspects related to bone quality and highlighting the visceral fat as an active tissue. The use of detailed variables, including some having a practical and helpful approach—like the lateral chest X‐ray—may be considered. Although our cohort includes patients only without treatment and then well selected before any bias, we need to report a limited sample size. In this context, we report a limitation related to high prevalence in the iVAT group of obese (81%) and male (87%) patients: this has limited our consideration of the impact of VAT as an active tissue regardless of obesity and gender.

## 5 CONCLUSION

In untreated patients with mild‐to‐severe OSA, the VAT impacts bone quality parameters of the lumbar spine (TBS). The impact on the bone can also result in asymptomatic vertebral fractures. In patients with OSA, this metabolically active tissue needs to be monitored, and provides one more reason for it to be managed with dieting or surgery.

## AUTHOR CONTRIBUTIONS

**Giulia Sartori:** Conceptualization; methodology; writing – original draft; data curation. **Francesco Bertoldo:** Conceptualization; writing – review and editing; methodology; data curation. **Andrea Gretter:** Conceptualization; data curation. **Federica Margherita Lovati:** Conceptualization; data curation. **Rosaria Caprino:** Conceptualization; data curation. **Giovanni Viterale:** Conceptualization; data curation. **Ernesto Crisafulli:** Conceptualization; writing – original draft; writing – review and editing; methodology; formal analysis; supervision.

## FUNDING INFORMATION

This study received no specific grant from any funding agency in the public, commercial or not‐for‐profit sectors.

## CONFLICT OF INTEREST STATEMENT

All authors declare to have no conflicts of interest related to the manuscript.

## Data Availability

The data that support the findings of this study are available from the corresponding author upon reasonable request.
